# Clinical Profiles and Prognoses of Adult Patients with Full-Frequency Sudden Sensorineural Hearing Loss in Combination Therapy

**DOI:** 10.3390/jcm12041478

**Published:** 2023-02-13

**Authors:** Yuanping Zhu, Sihai He, Kang Liao, Meihua Li, Zhibin Zhao, Hongyan Jiang

**Affiliations:** Otolaryngology-Head and Neck Surgery Hospital of Hainan Province, Hainan General Hospital (Hainan Affiliated Hospital of Hainan Medical University), Haikou 570311, China

**Keywords:** sudden sensorineural hearing loss, full-frequency, prognosis, vertigo, body mass index

## Abstract

We aimed to characterize the clinical profiles and short-term outcomes of adult patients with full-frequency idiopathic sudden sensorineural hearing loss (ISSNHL) treated uniformly with combination therapy, and to determine the prognostic predictors for the combination therapy. A total of 131 eligible cases hospitalized in our department from January 2018 to June 2021 were retrospectively reviewed. All enrolled cases received a standardized combination therapy employing intravenous methylprednisolone, batroxobin, and *Ginkgo biloba* extract during the 12 days of hospitalization. The clinical and audiometric profiles were compared between recovered patients and their unrecovered counterparts. The overall recovery rate was 57.3% in the study. Accompanying vertigo (odds ratio = 0.360, *p* = 0.006) and body mass index (BMI, odds ratio = 1.158, *p* = 0.016) were two independent predictors of hearing outcomes of the therapy. The male gender and cigarette-smoking history were marginally associated with good hearing prognosis (*p* = 0.051 and 0.070, respectively). Patients with BMI ≥ 22.4 kg/m^2^ had a better chance of hearing recovery (*p* = 0.02). Conclusions: Accompanying vertigo and low BMI (<22.4 kg/m^2^) were independently associated with poor prognosis for full-frequency ISSNHL in combination therapy. Male gender and cigarette-smoking history might be considered positive effects on hearing prognosis.

## 1. Introduction

Sudden sensorineural hearing loss (SSNHL), defined as a rapid onset of sensorineural hearing loss of no less than 30 dB affecting at least three consecutive frequencies on an audiogram between 250 and 8000 Hz within 72 h [1], is a typical otolaryngological emergency requiring prompt diagnosis and treatment. The prevalence is estimated at 5 to 27 per 100,000 in the United States [1], and is rising in China. A recent investigation estimated that the annual incidence of SSNHL in mainland China is 19 per 100,000 [2]. More than 90% of SSNHL is considered idiopathic with no identifiable causes. Several mechanisms have been proposed to address the pathogenesis of idiopathic SSNHL (ISSNHL), including viral attack, cochlear ischemia, and autoimmune disorders [3]. Corticosteroids are universally accepted and prescribed as the first-line treatment for ISSNHL [1,4,5]. Other therapies, such as hyperbaric oxygen therapy (HBOT), antivirals, vasoactive agents including prostaglandin E1, *Ginkgo biloba* extract, and nerve growth factors (NGFs), are also selectively used among institutions [6,7,8,9,10]. Unfortunately, none of these therapies has been proven to be effective for all cases. Since ISSNHL is a multifactorial disorder, the ideal management of it requires an individualized treatment strategy.

According to the affected frequencies on the audiogram, ISSNHL can be classified as low-frequency (250, 500, and 1000 Hz), high-frequency (2000, 4000, and 8000 Hz), and full-frequency (250, 500, 1000, 2000, 4000, and 8000 Hz), with different underlying etiology [4]. Vascular occlusion has been reckoned to be a significant cause of full-frequency ISSNHL. The cochlea is fed only by a single tenuous end artery and has no collateral circulation. Thus, it is very susceptible to damage by vascular blockage, resulting in cochlear ischemia and full-frequency hearing loss [11]. In China, combination therapy employing corticosteroids, batroxobin, and *Ginkgo biloba* extract has been recommended as the first-line option in treating full-frequency ISSNHL [4]. Corticosteroids have powerful inhibitory effects on the inflammatory cell-death cascade in ISSNHL. The extract of *Ginkgo biloba* 761 (EGb 761), containing approximately 24% flavonoid and 6% terpenes lactones, could improve cochlear blood flow by promoting vasodilation. It also acts as an antioxidant to attenuate oxidative stress provoked in ISSNHL. Additionally, batroxobin is a thrombin-like serine protease isolated from the venom of the snakes *Bothrops atrox* and *B. moojeni*. It is applied in clinical treatment to the management of thrombotic conditions as a defibrinogenating agent [12]. However, the hearing outcomes and potential prognostic factors of the therapy remain debatable.

In this study, we aimed to report the clinical profiles and short-term hearing outcomes of patients with full-frequency ISSNHL receiving combination therapy and to determine the prognostic factors associated with hearing improvements.

## 2. Materials and Methods

### 2.1. Subjects

Adult patients diagnosed with unilateral full-frequency ISSNHL admitted to our department from January 2018 to June 2021 were enrolled in the study. The medical records were retrospectively investigated. Eligible subjects must not have had either a prior episode of SSNHL or a history of Ménière’s disease or other cochlear lesions, and must have received prompt treatment within two weeks of symptom onset. Otoscopy and audiometric examinations were performed in all involved patients, including pure tone and speech audiometry, tympanometry, distortion product otoacoustic emission (DPOAE), and auditory brainstem response (ABR), to confirm the diagnosis of ISSNHL. Magnetic resonance imaging (MRI) of the brain and internal auditory canals was also conducted to evaluate the presence of retrocochlear pathology. Those with middle ear diseases or abnormal MRI findings, such as labyrinthine hemorrhage, vestibular schwannoma, or other cerebellopontine angle tumors, were excluded. Patients with comorbidities of hypertension or diabetes, or taking any lipid-lowering drugs, anticoagulants, antiplatelet or fibrinolytic agents, were also excluded. Clinical data of all enrolled patients were collected and analyzed, including age, sex, laterality, the time to initial treatment, the severity of hearing loss, accompanying tinnitus, vertigo, and aural fullness, and cigarette-smoking history. Peripheral blood samples were obtained on admission for basic blood tests and biochemistry studies.

### 2.2. Treatment

All enrolled patients received a standardized combination therapy during the 12 days of hospitalization. Methylprednisolone was dripped intravenously at 120 mg/d for the first four days, followed by a 40 mg taper every four days. Up to five shots of alternate-day batroxobin were given intravenously at 10 Bu for the first shot, then reduced to 5 Bu for the following shots. The plasma fibrinogen (Fg) level was closely monitored before each shot. Once the Fg level fell below 1.0 g/L, batroxobin injection was skipped to the next day to avoid the risk of hemorrhage. Additional *Ginkgo biloba* extract EGb 761 was infused intravenously at 87.5 mg/d.

### 2.3. Audiometric Evaluation

Hearing thresholds were determined by measuring air conduction pure tone average (PTA) at 250, 500, 1000, 2000, 4000, and 8000 Hz. According to the revised grading system recently released by World Health Organization (WHO) [13], the severity of hearing loss was graded as mild (20 to <35 dB HL), moderate (35 to <50 dB HL), moderately severe (50 to <65 dB HL), severe (65 to <80 dB HL), profound (80 to <95 dB HL), or complete (≥95 dB HL) based on PTA. Hearing gains were assessed by comparing the initial PTA on admission with the follow-up PTA 2 weeks after discharge. Hearing outcomes were evaluated according to the guideline proposed by the Chinese Society of Otorhinolaryngology-Head and Neck Surgery (CSOHNS) [4]. Complete recovery required a hearing level within 25 dB, or equivalent to that of the unaffected ear. Marked recovery was defined as a hearing gain of 30 dB or more. A hearing gain of 15 to <30 dB was considered partial recovery. Any hearing gain of less than 15 dB was classified as no recovery. The overall recovery rate was calculated as the sum of all complete, marked, and partial recovery rates. For statistical analysis, patients were subclassified into two groups based on their hearing outcomes: one consisting of those with complete, marked, and partial recovery, and the other with no recovery.

### 2.4. Statistical Analysis

Statistical analysis was performed using SPSS 22.0 software. The independent samples *t*-test was carried out to compare the means of metric variables between two groups when complying with a normal distribution. Otherwise, non-parametric tests were used. The Pearson *χ*^2^ test was conducted to compare frequencies of categorical variables between different groups. Spearman’s rank correlation coefficient *𝜌* was used when one variable had a continuous normal distribution and the other was non-normally distributed. Kendall’s *𝜏* was calculated to measure correlations between two non-normally distributed variables. In addition, multivariate logistic regression was employed to determine the independent prognostic factors on early hearing outcomes. The receiver operating characteristic (ROC) curve was further used to calculate the optimal cut-off points (also called the Youden Index) in the continuous variables predicting the hearing prognosis. Any *p*-value less than 0.05 was considered to be of statistical significance.

## 3. Results

- **Clinical and audiometric profiles of the patients and their short-term hearing outcomes**

A total of 131 eligible patients were enrolled in the study, consisting of 74 (56.5%) males and 57 (43.5%) females, aged between 19 and 64 years old, with a median age of 47. Their clinical profiles are presented in Table 1.

Over 90% of enrolled patients had severe to complete hearing loss regarding their initial PTA. No adverse events were observed during treatment. Hearing outcomes were presented as follows according to CSOHNS criteria: 9 (6.9%) patients made a complete recovery; 23 (17.6%), a marked recovery; 43 (32.8%), a partial recovery; and 56 (42.7%), no recovery. The overall recovery rate of the enrolled patients was 57.3% in our study. The linear-by-linear association indicated no significant trend for hearing recovery with the alleviating severity of hearing loss (*χ*^2^ = 0.593, d*f* = 1, *p* = 0.441).

Univariate analysis demonstrated a higher recovery rate in male patients compared to female counterparts (*χ*^2^ = 5.584, d*f* = 1, *p* = 0.018). Similarly, patients with cigarette-smoking history revealed a better hearing prognosis (*χ*^2^ = 4.330, d*f* = 1, *p* = 0.037). Furthermore, accompanying vertigo significantly reduced the hearing recovery rate (*χ*^2^ = 8.134, d*f* = 1, *p* = 0.004). Other factors, including age, affected side, time to initial treatment, initial PTA, and presence of associated tinnitus and aural fullness seemed to have little impact on the hearing prognosis (*p* > 0.05).

- **Responses of plasma fibrinogen to the combination therapy**

**Figure 1:**
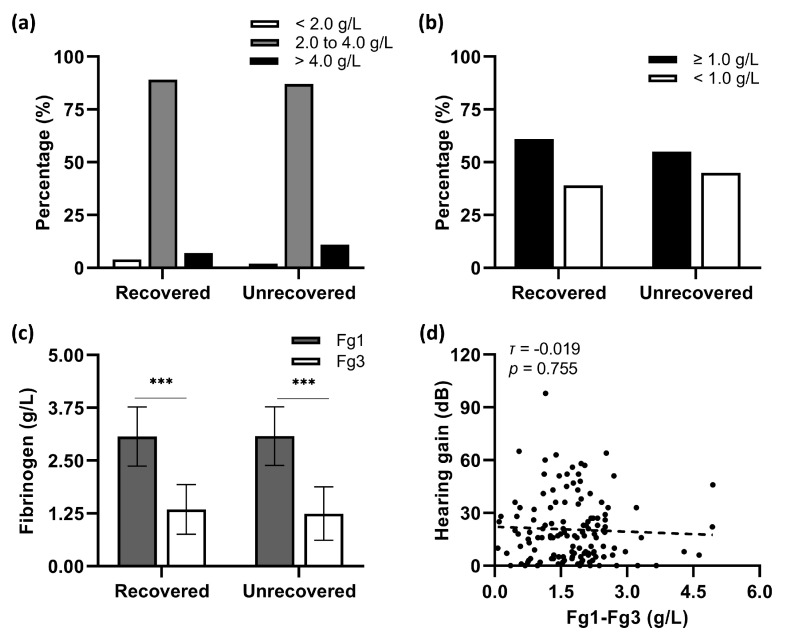
Comparison of responses of plasma fibrinogen to the combination therapy between patients with and without hearing recovery. (**a**) A share of 89.3% of patients with hearing recovery presented a normal value (2.0 to 4.0 g/L) of Fg1. Pearson *χ*^2^ test demonstrated an equal proportion in unrecovered patients (87.5%). (**b**) The proportion of Fg3 above or below the critical value (<1.0 g/L) was not statistically associated with hearing outcomes. (**c**) Wilcoxon matched-pair signed-rank test revealed that fibrinogen levels significantly decreased after the first dose of batroxobin in all involved patients. However, there were no statistical differences in Fg1 and Fg3 between patients with opposite hearing outcomes. Data were presented as means ± SD. *** *p* < 0.001. (**d**) Kendall’s correlation analysis indicated no significant correlation between patients’ hearing improvement and the reduction of fibrinogen levels after batroxobin application.

Nearly 90% of involved patients demonstrated a normal value (2.0 to 4.0 g/L) of baseline plasma fibrinogen levels (Fg1) before the first shot of 10-Bu batroxobin (Figure 1a). The average Fg1 levels in hearing-recovered patients were not significantly different from those in their unrecovered counterparts (*T*_1_ = 66.36, *n*_1_ = 56; *T*_2_ = 65.73, *n*_2_ = 75; *p* = 0.926, Figure 1c). As shown in Figure 1b, the fibrinogen levels measured before the second batroxobin injection (Fg3) fell below the critical value (<1.0 g/L) in 38.7% of patients with recovery and 44.6% without hearing recovery (*χ*^2^ = 0.473, d*f* = 1, *p* = 0.492). The Mann–Whitney *U* test indicated a similar reduction in the fibrinogen levels between the two groups (*T*_1_ = 69.17, *n*_1_ = 56; *T*_2_ = 63.63, *n*_2_ = 75; *p* = 0.409, Figure 1c). No statistical correlation was found between hearing gains and the reduction of fibrinogen levels (Fg1–Fg3) in this study (Kendall’s *τ* = −0.019, *p* = 0.755; Figure 1d).

- **Correlation of body mass index (BMI) and serum lipid levels with hearing outcomes of the combination therapy**

**Figure 2:**
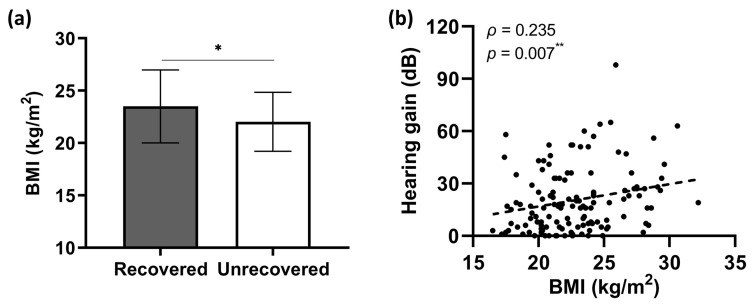
The relationship between BMI and hearing outcomes by combination therapy. (**a**) Two independent samples *t*-test indicated significantly greater BMI points in patients with hearing recovery (23.5 ± 3.48 kg/m^2^) compared with the unrecovered counterparts (22.0 ± 2.82 kg/m^2^). Data are presented as means ± SD. (**b**) Spearman’s correlation analysis revealed a positive correlation of BMI with hearing gain by combination therapy. * *p* < 0.05, ** *p* < 0.01.

Figure 2a compares average BMI points between patients with opposite hearing outcomes, showing statistically greater points in the recovered group than in the unrecovered one (*t* = 2.601, d*f* = 129, *p* = 0.010). Furthermore, the BMI points were positively correlated with hearing gains in the presented study (Spearman’s *ρ* = 0.235, *p* = 0.007; Figure 2b).

**Figure 3:**
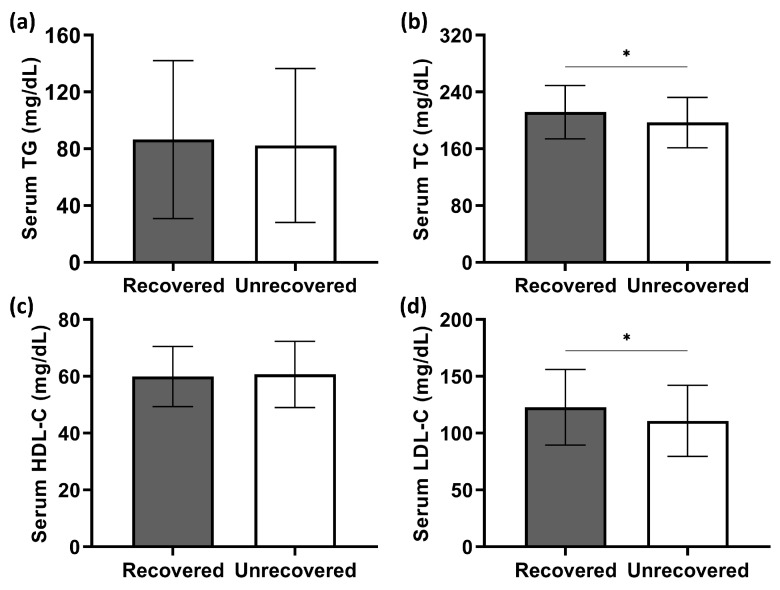
Comparison of serum TG (**a**), TC (**b**), HDL-C (**c**), and LDL-C (**d**) concentrations between recovered and unrecovered patients. Two independent samples *t*-test demonstrated that serum TC and LDL-C concentrations were significantly higher in recovered patients. Data are presented as means ± SD. * *p* < 0.05.

**Figure 4:**
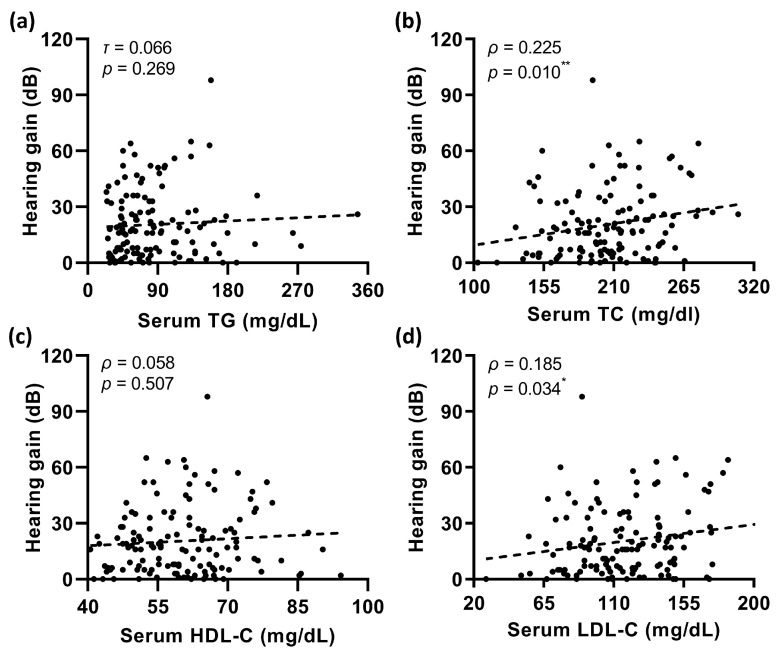
Correlation analysis of patients’ hearing gains with serum TG (**a**), TC (**b**), HDL-C (**c**), and LDL-C (**d**) levels showed that higher serum TC and LDL-C concentrations were correlated with more hearing gains. * *p* < 0.05, ** *p* < 0.01.

Patients’ serum lipid levels are shown in Figure 3. The Shapiro–Wilk test indicated that only triglyceride (TG) concentrations were not normally distributed (*p* < 0.001). Recovered patients were similar to their unrecovered counterparts in terms of serum TG levels (*T*_1_ = 62.58, *n*_1_ = 56; *T*_2_ = 68.55, *n*_2_ = 75; *p* = 0.373, Figure 3a). In contrast, the serum concentrations of total cholesterol (TC) and low-density lipoprotein cholesterol (LDL-C) in patients with hearing recovery were significantly higher than in those without recovery (*p* = 0.026 and 0.037, respectively; Figure 3b,d). There was no statistical difference in serum high-density lipoprotein cholesterol (HDL-C) levels between the two groups (*t* = −0.378, d*f* = 129, *p* = 0.706; Figure 3c). Correlation analysis further revealed that higher serum TC concentrations correlated with greater hearing gains (Spearman’s *ρ* = 0.225, *p* = 0.010; shown in Figure 4b). Similarly, a significantly positive correlation of serum LDL-C levels was presented with hearing gains (Spearman’s *ρ* = 0.185, *p* = 0.034; shown in Figure 4d).

- **The multivariate logistic regression model of possible prognostic predictors**

**Figure 5:**
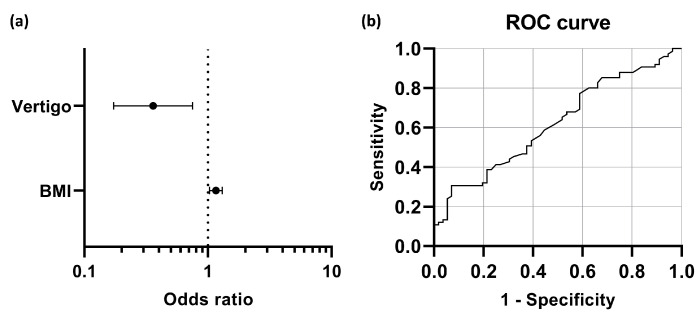
Independent predictive factors for hearing prognosis. (**a**) The forest plot illustrates the relative importance of concurrent vertigo and BMI in predicting the hearing prognosis in combination therapy. (**b**) The ROC curve reveals that BMI was useful in predicting the hearing outcome after treatment. The area under the curve (AUC) was 0.619 (95% CI = 0.523 to 0.715, *p* = 0.02).

In order to determine which noticeable variables independently predict the hearing outcome of the combination therapy, the multivariate logistic regression model was conducted, including parameters that achieved *p* < 0.05 in the univariate analysis (gender, presence of associated vertigo, cigarette-smoking, BMI, TC, and LDL-C; details shown in Table 2). The result indicated that accompanying vertigo and BMI were significant predictors of early hearing outcomes of the therapy (*p* = 0.006 and 0.016, respectively). The male gender and cigarette-smoking history also seemed to be associated with a good hearing prognosis; however, their statistical significance levels were marginal (*p* = 0.051 and 0.070, respectively). As shown in Figure 5a, the odds of patients with concurrent vertigo achieving hearing recovery were 0.360 times those of their counterparts without vertigo. For each unit increase in patients’ BMI, the chances of hearing recovery were 1.158 times higher. The ROC curve illustrated that the optimal cut-off point (the Youden Index) in BMI was 22.4 km/m^2^, predicting that patients with BMI ≥ 22.4 km/m^2^ tended to have hearing recovery after the therapy (shown in Figure 5b).

**Figure 6:**
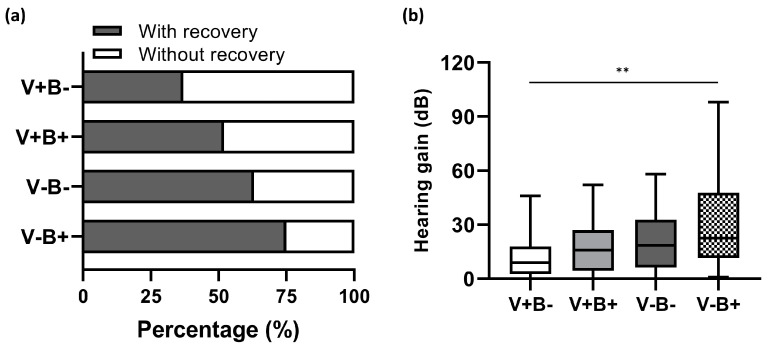
Comparison of hearing outcomes among patients with disparate prognostic predictors. (**a**) The overall hearing recovery rates varied statistically among patients with different prognostic factors. V+B- stands for dizzy patients with BMI < 22.4 kg/m^2^, V+B+ for dizzy patients with BMI ≥ 22.4 kg/m^2^, V-B- for patients with no accompanying vertigo plus BMI < 22.4 kg/m^2^, and V-B+ for patients with no accompanying vertigo plus BMI ≥ 22.4 kg/m^2^. (**b**) The more predictors of good prognoses the patients possessed, the better the hearing improvements that were gained. The box boundaries indicate upper and lower quartiles (*Q*_U_ and *Q*_L_). The line within the boxes marks the median (Mdn). The whiskers below and above the box represent the minimum and maximum. Data are presented as Mdn (IQR). ** *p* < 0.01.

Furthermore, we examined how well these two independent prognostic factors predict hearing outcomes. Based on their status of vertigo (V+, presence of vertigo; V-, absence of vertigo) and BMI (B+, BMI ≥ 22.4 kg/m^2^; B-, BMI < 22.4 kg/m^2^), all the enrolled patients were subdivided into four groups: V+B+, V+B-, V-B+, and V-B-. The overall recovery rate reached 75% (27/36) in the group of V-B+, but decreased to 62.5% (20/32) and 51.5% (17/33), respectively, in V-B- and V+B+. The lowest recovery rate of 36.7% (11/30) was found in V+B- (shown in Figure 6a). The Pearson *χ*^2^ test indicated significant differences among patients with disparate predictors for hearing prognosis (*χ*^2^ = 10.632, d*f* = 3, *p* = 0.014). In parallel with this, V-B+ patients had the greatest hearing gain of 22.5 (36) dB, while the least hearing gain of 9.0 (15) dB was observed in V+B- counterparts (*H* = 13.201, d*f* = 3, *p* = 0.004; shown in Figure 6b).

## 4. Discussion

Several therapeutic options have been tried based on the various mechanisms proposed for the pathogenesis of ISSNHL, but none guarantee effectiveness. In this study, we paid particular attention to the triad combination therapy employing corticosteroid, defibrinogenation agent batroxobin, and *Ginkgo biloba* extract, which is recommended in China for full-frequency cases [4]. The short-term hearing outcomes and prognostic factors of the treatment were investigated.

About 57.3% of the enrolled patients achieved hearing recovery to some extent. Our study’s overall recovery rate makes sense compared to other studies. Sun et al. [14] assessed the hearing outcomes one week after the initial treatment and reported a total recovery rate of 68.9% (73/106) in adult patients aged between 18 and 65 years. By contrast, Lee et al. [15] reported an even lower hearing recovery rate (40%) two to three months after onset in patients with profound hearing loss. The following reasons should be considered to explain the difference in hearing recovery rates among studies. It has been well-documented that hearing recovery negatively correlates with the severity of initial hearing loss [1,16,17,18]. Correspondingly, over 90% (118/131) of the enrolled patients in our study suffered from severe to complete deafness before treatment, which reduced the chance of hearing recovery. Additionally, the definition of hearing recovery varies among studies [1,14,15], which makes the comparison of effective rates less meaningful.

All enrolled patients in the study were between 19 and 64 years old, with a similar number of men and women. Univariate analysis suggested that gender, cigarette-smoking history, presence of associated vertigo, BMI, and serum TC and LDL-C levels may interfere with early hearing outcomes of the combination therapy, which, with the exception of vertigo, are also certified risk factors for arteriosclerotic cardiovascular disease (ASCVD) [19,20]. The multivariate logistic regression model further confirmed concurrent vertigo and BMI as two independent predictors for hearing prognosis in the study. It also implied that the male gender and cigarette-smoking history were marginally associated with hearing recovery.

The majority of patients (59.5%) were middle-aged (46 to 65 years old); thus, age had little impact on the hearing outcomes in this study. Interestingly, a significantly higher hearing recovery rate was found in male patients than in female ones, and there was a more significant proportion of smoking patients in the recovered group. Multivariate analysis further revealed that both the male gender and cigarette-smoking history were marginally associated with a good hearing prognosis. Such findings have seldom been reported in the literature. There was an equal prevalence of ISSNHL between the left and right ears. No significant association was found between hearing recovery and affected sides, which aligns with the literature [16,21]. All the enrolled patients received prompt therapy within 14 d of symptom onset, ensuring the intervals between symptom onset and initial treatment barely affected the hearing outcomes in the study, which is in accordance with preceding reports [16,17].

The presence of vertigo at the time of symptom onset was proven to be an independent predictor for poor hearing prognosis in the presented study, which matches the finding from previous studies. Possible theories include occlusion of the internal auditory artery, labyrinthine membrane rupture, and concurrence of vestibular neuritis. Tinnitus was nearly a universal accompanying complaint among patients, and about half of the patients reported a feeling of ear fullness. There was no significant association of hearing recovery with either tinnitus or aural fullness. All these findings were already well-known [16,22].

Prior studies have stated that high BMI and serum TC concentration correlate with poor hearing recovery in corticosteroid therapy [23,24]. However, contrary findings were observed in our study. Univariate analysis revealed significantly larger BMI and higher TC and LDL-C levels in patients with hearing recovery than in their unrecovered counterparts when treated using the combination regimen. Furthermore, hearing improvement was positively correlated with BMI, TC, and LDL-C. However, the multivariate analysis only identified BMI as an independent predictor for hearing prognosis. We theorize that the relatively small sample size, slight difference, and wide variation weakened the statistical power of the other two variables, TC and LDL-C.

The discrepancy unveiled in our study could be partly explained by the cochlear microcirculation thrombosis theory. Theoretically, atherosclerosis, which induces vascular occlusion of coronary and cerebral arteries resulting in angina, myocardial infarction, and stroke, could play a similar role in blocking the terminal arteries supplying the cochlea, thus interrupting cochlear perfusion and eventually resulting in full-frequency ISSNHL [25]. Obesity has been reckoned as an additional ASCVD risk factor by the American Association of Clinical Endocrinologists (AACE). Furthermore, high TC and LDL-C levels are two independent major risk factors for ASCVD [19]. Hence ISSNHL patients with larger BMI or higher TC/LDL-C levels have a greater chance of atherosclerotic thrombogenesis in the cochlea. Fibrinogen has also been found to be essential in developing atherosclerosis [26]. Previous in vivo animal experiments revealed that reducing fibrinogen levels significantly improved cochlear microcirculation and hearing loss [27]. In the presented study, the extra defibrinogenation agent batroxobin was employed in the combination therapy, and could effectively reduce atherosclerosis by remarkably lowering the fibrinogen levels, resulting in cochlear microcirculation improvement. Therefore, the larger the BMI, the greater the possibility of cochlear atherosclerosis, and the more influential the combination therapy. Similar explanations apply to our findings, mentioned above, that the male gender and cigarette-smoking history had a borderline positive impact on hearing outcomes in the study. Both gender and smoking are explicitly listed as risk factors for ASCVD in the latest guidelines for dyslipidemia management proposed by the European Society of Cardiology (ESC) and European Atherosclerosis Society (EAS). Male smokers are at much higher risk for ASCVD than female non-smokers under the same conditions of age, systolic blood pressure, and TC level [20]. The presented study provides further support for the hypothesis that microvascular attack may play a critical role in the pathogenesis of full-frequency ISSNHL.

Although this study is limited by its retrospective design, short observational window, and relatively small sample size, it is the first clinical research addressing the correlation of the short-term hearing outcomes of the triad combination therapy for full-frequency ISSNHL with atherosclerotic risk factors in Chinese patients, and suggesting that combination therapy could be recommended for full-frequency ISSNHL patients with a high BMI (≥ 22.4 km/m^2^). The findings from our study provide preliminary clinical guidance for physicians to make individualized treatment decisions for patients with full-frequency ISSNHL and support the theory of cochlear microcirculation disturbance as a vital cause of ISSNHL.

## 5. Conclusions

Our study identified the presence of concurrent vertigo and low BMI (<22.4 kg/m^2^) as two independent prognostic factors for poor hearing outcomes in combination therapy employing methylprednisolone, batroxobin, and *Ginkgo biloba* extract. Additionally, male patients and those with cigarette-smoking history may also tend to have better hearing outcomes after combination treatment.

## Figures and Tables

**Table 1:** Comparison of clinical profiles between patients with and without hearing recovery ^†^.

Parameters		Hearing Outcomes, N (%)		
	Patients, N (%)	With Recovery	Without Recovery	*p*-Value
Total	131 (100)	75 (57.3)	56 (42.7)	
Gender				0.018 *
Male	74 (56.5)	49 (66.2)	25 (33.8)	
Female	57 (43.5)	26 (45.6)	31 (54.4)	
Age (y)	47 (19)	48 (20)	46 (19)	0.957
Affected side				0.705
Left	75 (57.3)	44 (58.7)	31 (41.3)	
Right	56 (42.7)	31 (55.4)	25 (44.6)	
Time to initial treatment (d)	4 (4)	5 (4)	4 (6)	0.798
Initial PTA (dB HL)	87 (29)	84 (29)	91 (34)	0.255
Final PTA (dB HL)	72 (34)	54 (46)	86 (29)	0.000 ***
Severities of hearing loss				0.441
Moderately severe	13 (9.9)	8 (61.5)	5 (38.5)	
Severe	32 (24.4)	19 (59.4)	13 (40.6)	
Profound	36 (27.5)	22 (61.1)	14 (38.9)	
Complete	50 (38.2)	26 (52.0)	24 (48.0)	
Associated symptoms				
Tinnitus	126 (96.2)	73 (57.9)	53 (42.1)	0.738
Vertigo	63 (48.1)	28 (44.4)	35 (55.6)	0.004 **
Aural fullness	89 (67.9)	51 (57.3)	38 (42.7)	0.986
Cigarette smoking	22 (16.8)	17 (77.3)	5 (22.7)	0.037 *

^†^ Mann–Whitney *U* test was applied to age, time to initial treatment, and initial and final PTA, which are presented as Mdn (IQR). Linear-by-linear association was tested for the severity of hearing. Continuity correction test was used for tinnitus. Other variables were analyzed with Pearson *χ*^2^ test. Abbreviation: Mdn, median; IQR, inter-quartile range. * *p* < 0.05, ** *p* < 0.01, *** *p* < 0.001.

**Table 2:** The logistic regression for the relationship between hearing recovery and possible predictors.

Variable	Coefficient	S.E.	Wald *χ*^2^	*p*-Value	OR	95%CI
Vertigo	−1.022	0.374	7.463	0.006 **	0.360	0.173 to 0.749
BMI	0.146	0.061	5.776	0.016 *	1.158	1.027 to 1.305
Constant	−2.528	1.394	3.288	0.070	0.080	

Abbreviation: S.E., standard error; OR, odds ratio; CI, confidential interval. * *p* < 0.05, ** *p* < 0.01.

## Data Availability

The data presented in this study are available on request from the corresponding author.
